# Massachusetts Veterinary Association

**Published:** 1890-12

**Authors:** Austin Peters

**Affiliations:** Secretary


*Massachusetts Veterinary Association.*—The regular monthly meeting of the Massachusetts Veterinary Association was held at 19 Boylston Place, Boston, October 22d, 1890. The president, Thos. Blackwood, was in the chair. Members present: Drs. Blackwood, Banker, Howard, Marshall, Peterson, Winchester and the secretary. Honorary member, Dr. Stickney.

Routine business was transacted, after which, there being no paper presented, the different members present took part in a general discussion of various topics of interest.

Meeting adjourned. AUSTIN PETERS, *Secretary.*

